# REducing unwarranted variation in the Delivery of high qUality hip fraCture services in England and Wales (REDUCE): protocol for a mixed-methods study

**DOI:** 10.1136/bmjopen-2021-049763

**Published:** 2021-05-19

**Authors:** Rita Patel, Sarah Drew, Antony Johansen, Tim Chesser, Muhammad K Javaid, Xavier L Griffin, Tim Jones, Jill Griffin, Marianne Bradshaw, Katie Whale, Estela Capelas Barbosa, Elsa M R Marques, Yoav Ben-Shlomo, Rachael Gooberman-Hill, Andrew Judge, Celia L Gregson

**Affiliations:** 1 Musculoskeletal Research Unit, Translational Health Sciences, Bristol Medical School, University of Bristol, Bristol, UK; 2 Division of Population Medicine, School of Medicine, Cardiff University and University Hospital of Wales, Cardiff, UK; 3 National Hip Fracture Database, Royal College of Physicians, London, UK; 4 Department of Trauma and Orthopaedics, Southmead Hospital, North Bristol NHS Trust, Bristol, UK; 5 Nuffield Department of Orthopaedics, Rheumatology and Musculoskeletal Sciences, University of Oxford, Oxford, UK; 6 Division of Orthopaedics, Barts and The London School of Medicine and Dentistry, Queen Mary University of London, London, UK; 7 Royal London Hospital, Barts Health NHS Trust, London, UK; 8 Clinical & Operations Directorate, Royal Osteoporosis Society, Bath, UK; 9 NIHR Biomedical Research Centre at University Hospitals Bristol and Weston NHS Foundation Trust and the University of Bristol, Bristol, UK; 10 Population Health Sciences, Bristol Medical School, University of Bristol, Bristol, UK

**Keywords:** hip, geriatric medicine, qualitative research, health economics

## Abstract

**Introduction:**

Substantial variation in the delivery of hip fracture care, and patient outcomes persists between hospitals, despite established UK national standards and guidelines. Patients’ outcomes are partly explained by patient-level risk factors, but it is hypothesised that organisational-level factors account for the persistence of unwarranted variation in outcomes. The mixed-methods REducing unwarranted variation in the Delivery of high qUality hip fraCture services in England and Wales (REDUCE) study, aims to determine key organisational factors to target to improve patient care.

**Methods and analysis:**

Quantitative analysis will assess the outcomes of patients treated at 172 hospitals in England and Wales (2016–2019) using National Hip Fracture Database data combined with English Hospital Episodes Statistics; Patient Episode Database for Wales; Civil Registration (deaths) and multiple organisational-level audits to characterise each service provider. Statistical analyses will identify which organisational factors explain variation in patient outcomes, and typify care pathways with high-quality consistent patient outcomes. Documentary analysis of 20 anonymised British Orthopaedic Association hospital-initiated peer-review reports, and qualitative interviews with staff from four diverse UK hospitals providing hip fracture care, will identify barriers and facilitators to care delivery. The COVID-19 pandemic has posed a major challenge to the resilience of services and interviews will explore strategies used to adapt and innovate. This system-wide understanding will inform the development, in partnership with key national stakeholders, of an ‘Implementation Toolkit’ to inform and improve commissioning and delivery of hip fracture services.

**Ethics and dissemination:**

This study was approved: quantitative study by London, City and East Research Ethics Committee (20/LO/0101); and qualitative study by Faculty of Health Sciences University of Bristol Research Ethics Committee (Ref: 108284), National Health Service (NHS) Health Research Authority (20/HRA/71) and each NHS Trust provided Research and Development approval. Findings will be disseminated through scientific conferences, peer-reviewed journals and online workshops.

**Strengths and limitations of this study**

- A mixed-methodology approach will aid identification of hospital-level organisational factors which explain adverse patient outcomes following hip fracture, and which are amenable to improvement across the UK.
- This study is novel in terms of its scale and the unique datasets used, which gives a rare opportunity to robustly assess a complex system of care and the impacts this system has on patients with hip fracture and National Health Service (NHS) budgets.
- Use of quantitative, economic and qualitative analysis will provide a system-wide understanding of the hip fracture care pathway, which will inform development of an Implementation Toolkit, in partnership with key national stakeholders, to improve future service design.
- While multiple organisational datasets exist relevant to patient care in NHS hospitals, linking this NHS activity to comprehensive social care data is not viable, hence, social care sequalae following hip fracture admission does not form part of this protocol.
- Currently the NHS is experiencing unprecedented pressures; the REducing unwarranted variation in the Delivery of high qUality hip fraCture services in England and Wales study will determine the most efficient management pathways for high cost patients, to improve patient outcomes and free NHS resources for use elsewhere.

## Introduction

Each year in the UK more than 70 000 older adults sustain a fragility fracture of the hip.^1^ Such fractures are indicative of osteoporosis.^1^ Hip fractures are costly to patients, relatives and the National Health Service (NHS), with a significant impact on quality of life^2^; a quarter of patients die within 1 year of hip fracture.^3^ Research has shown annual NHS medical costs from hip fracture exceed £1.2 billion.^4^

Patients sustaining hip fractures almost invariably require an operation, but patient care has many complexities requiring contributions from various healthcare professionals at different time-points during an often-lengthy treatment journey. Many guidelines have been published trying to ensure all components of the care of patients sustaining hip fractures (hip fracture care) are provided consistently and to a high standard in all hospitals.^1 5–7^ While care has improved for some, there remains a great deal of variation across the UK in how health services deliver hip fracture care, so treatment still depends on where and when patients present to hospital. This unwarranted variation in care includes delays waiting for an operation, the type of operation performed, how much specialist help is provided, how soon physiotherapy is delivered, how thoroughly bone health is assessed, and more.

Substantial variation exists in how well patients recover after a hip fracture. Across the 172 hospitals currently providing hip fracture care in England and Wales, 1 month after hip fracture the proportion of patients who have died varies from 2% to 14% between hospitals,^8^ and the proportion who have been able to return home ranges from 29% to 85%.^9^ While overall 61% of patients are prescribed medication to reduce the chance of a further fracture in the future, this can vary enormously from 6% to 99.5% according to the hospital delivering hip fracture care.^10^ Time spent in hospital is highly variable (the median length of stay in acute and postacute NHS care is 17 days, IQR 10–30 days) and the chance of being readmitted to hospital within 30 days because of a deterioration after discharge is high at 16%.^11 12^ While patient outcomes are partly explained by patient-level risk factors (eg, age and comorbidity), it is hypothesised that organisational factors are responsible for variation in the delivery of fracture care pathways and hence patient outcomes; these organisational factors are potentially modifiable.

It is important to understand how the set-up and organisation of healthcare services affects patient recovery and outcomes after hip fracture. These services can vary in many ways, for example, types and grade of clinical staff; capacity to perform prompt operations; access to suitable rehabilitation services. It is expected these factors will explain variation in quality of care, patient outcomes and associated health costs. Understanding these will enable us to inform changes in healthcare services to minimise avoidable variation in fracture care and improve the quality of care for all patients across the country.

The 2020 COVID-19 pandemic has had an unprecedented impact on health service delivery across the NHS.^13 14^ Hospitals have needed to rapidly adapt and reorganise services to continue to deliver hip fracture care. To assist in service planning, NHS England^15 16^ and the British Orthopaedic Association (BOA)^17^ issued rapid guidance in March 2020 on the management of hip fracture patients during the coronavirus pandemic.^15–17^ The limited evidence to date suggests that there has been wide variation in how hospitals have reconfigured services^18 19^ and there is a lack of information about the impact of these changes on patient care.

The aim of this mixed-methods study is to determine the components of hospital service delivery of hip fracture care that predict patient outcomes post hip fracture, and the direct health costs attributable to these organisational factors. Furthermore, to understand factors that act as barriers and facilitators to the delivery of hip fracture care, including strategies that hospitals used to adapt and innovate hip fracture care delivery during the COVID-19 pandemic. Using these results, and working with key stakeholders, a toolkit will be developed, suitable for use by hospital managers, clinical leads and healthcare system leads across the country, to improve organisational delivery of high-quality hip fracture services. Understanding strategies hospitals used to reconfigure care during the COVID-19 pandemic will inform development of more resilient services in the future.

## Methods and analysis

### Quantitative study

#### Data sources: organisational level

**Figure 1:**
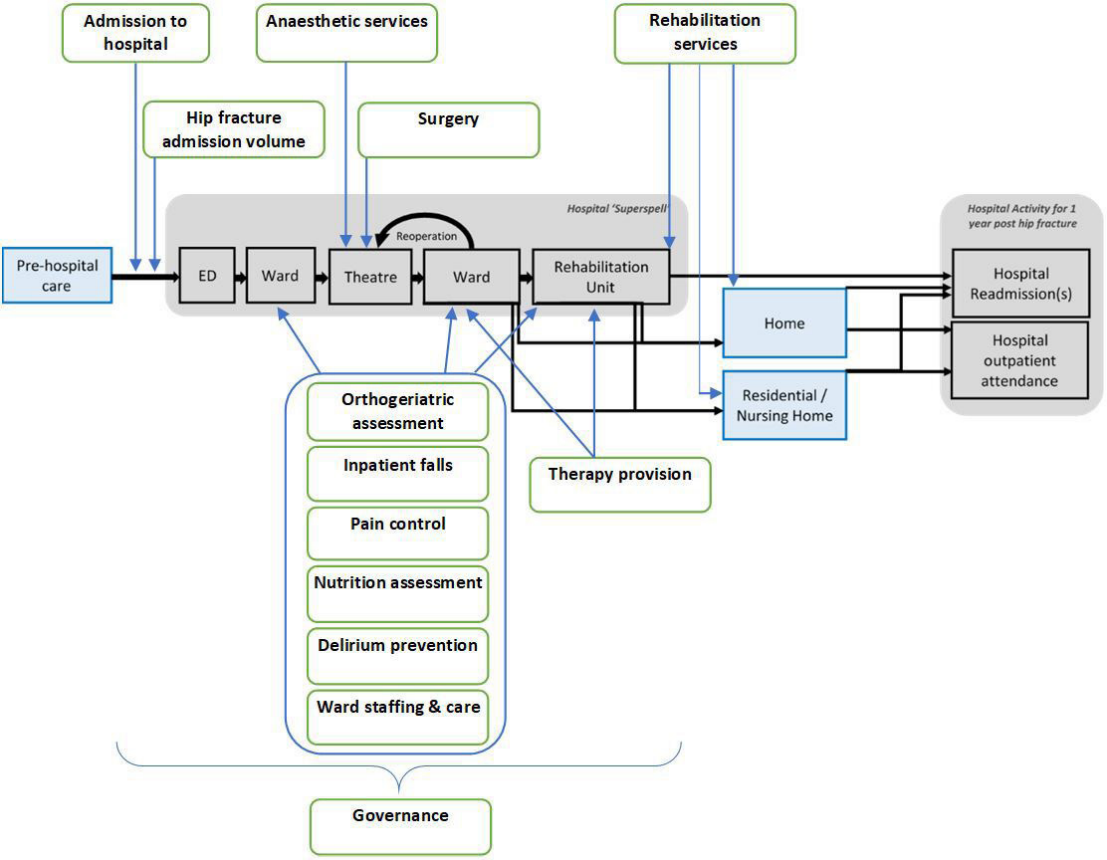
Hip fracture care pathway and domains of organisational-level data. Hip fracture care pathway flows from prehospital care, through the hospital superspell, through to any occurring hospital activity in the year after hip fracture. Organisational domains are indicated by green boxes, and these are mapped to the pathway.

Using a wide range of publicly available organisational-level service data, available at a hospital/trust provider level in England and Wales, including eight national audits and nine data series/ratings resources (table 1), data will be extracted to characterise each component of the hip fracture care pathway from admission to discharge (figure 1). The derived organisational metrics will be linked using hospital provider codes. Time-specific organisational metrics will be linked to patients by using the year in which they were admitted with their hip fracture. Organisational metrics aim to quantify provision of emergency, orthopaedic, anaesthetic, orthogeriatric, nursing, physiotherapy, rehabilitation and governance services.

**Table 1:** Organisational datasets included in the REducing unwarranted variation in the Delivery of high qUality hip fraCture services in England and Wales study

Organisational level dataset	England and Wales	Available years	Type of data available	Ref
NHFD Benchmark Summary	Combined	2016, 2017, 2018, 2019	Summary of hospital performance in three areas: assessment, surgery, outcomes	31
NHFD Best Practice	Combined	April 2016 to March 2019	The charts provide feedback on service quality and compliance with national care standards	31
NHFD Key Performance Indicator	Combined	December 2017 to March 2019	Describe the most important aspects of patient care	31
NHFD Charts (excluding BP and KPI)	Combined	April 2016 to March 2019	Charts with information on: Anaesthesia. Overall performance. Patient safety. Surgery used.	31
NHFD Facilities Audit/Survey	Combined	2016–17, 2017–2018, 2018–2019	Annual survey of facilities and performance of trauma units in England, Wales and Northern Ireland	8 9 44
National Audit of Inpatient Falls	Combined	2017 and 2019	Organisational audit Background. Policies, protocols and paperwork. Leadership and service provision. Clinical audit (snapshot of the care) Evidence of assessment and intervention in case notes. Observation at bedside/patient environment.	45 46
Physiotherapy Hip Fracture Sprint Audit	Combined	2017	Review of physiotherapy rehabilitation for hip fracture patients in the UK	47
Fracture Liaison Service Database	Combined	2016, 2017, 2018, 2019	National audit of secondary fracture prevention in England and Wales	48–50
Care Quality Commission	England	2016, 2017, 2018, 2019	CQC independent regulator of health and adult social care in England	51
NHS Staff Survey Themes	England	2016, 2017, 2018, 2019	Reports how NHS staff in England experience working for their respective NHS organisations	52
NHS Workforce Statistics	England	April 2016 to March 2019	Monthly numbers of NHS Hospital staff groups working in Trusts in England as headcount and full-time equivalents	53
NHS Bed Availability and Occupancy Data—Overnight	England	April 2016 to March 2019	A quarterly collection from all NHS organisations that operate beds, open overnight or day only. It collects the total number of available bed days and the total number of occupied bed days by consultant main specialty.	54
NHS Supporting Facilities Data Operating Theatres	England	April 2016 to March 2019	The number of operating theatres and supporting facilities in NHS organisations (trusts) in England	55
NHS Emergency Department Attendances and Emergency Admissions	England	April 2016 to March 2019	A&E attendances and emergency admission monthly statistics, NHS and independent sector organisations in England	56
NHS Staff	Wales	September 2016–2018, March 2019	Assignment count and full-time equivalent of directly employed NHS staff by grade and area of work	57
NHS Beds by Specialty: Availability and Occupancy Data	Wales	2016–2017, 2017–2018, 2018–2019	NHS Beds by organisation and specialty	58
NHS ED Attendances and Emergency Admissions	Wales	April 2016 to March 2019	Reports performance against waiting times targets by hospital. (Requested and received from NWIS directly, total emergency admissions.)	59

A&E, accident and emergency; BP, Best Practice; CQC, Care Quality Commission; ED, Emergency Department; KPI, Key Performance Indicator; NHFD, National Hip Fracture Database; NHS, National Health Service; NWIS, NHS Wales Informatics Service.

### Study population

#### Data sources: patient-level

Using routinely collected Hospital Episodes Statistics (HES) Admitted Patient Care (APC) data, that includes admissions to all English hospitals within the NHS (ie, excluding privately financed healthcare), patients will be linked by NHS Digital, the national health and social care data provider, to Office for National Statistics (ONS) Civil Registrations (deaths) mortality data for the same period. Similarly, in Wales the NHS Wales Informatics Service will link patients with hip fractures in the Patient Episode Database for Wales (PEDW) to ONS mortality data. The resulting HES-ONS and PEDW-ONS patients’ data extracts will then be linked to data from the UK’s National Hip Fracture Database (NHFD) for the admission. The NHFD, active since 2007, is a clinically led web-based audit of hip fracture care and secondary fracture prevention in England, Wales and Northern Ireland, data collected have informed the Best Practice Tariff (BPT) for hip fracture care since 2010.^20 21^

HES/PEDW provide information on patient demographics, admission, discharge, clinical diagnoses using International Classification of Diseases Tenth Revision (ICD-10) disease codes, and Classification of Interventions and Procedures version 4 codes.^22^ ONS mortality data are obtained from death certificates of all registered deaths in England and Wales,^23^ thus capturing deaths that occurred inside and outside of hospital. Each NHFD record includes information on patient demographics, anaesthetic risk grade, type of hip fracture and surgical operation performed.

Hip fracture admissions will be identified using ICD-10 codes for fractured neck of femur (S72.0), pertrochanteric fracture (S72.1), subtrochanteric fracture (S72.2) and unspecified fracture of femur (S72.9). The study population will consist of index cases of hip fracture (ie, the first occurrence of hip fracture), among English or Welsh residents (male and female) aged 60 years or more, admitted to an English or Welsh hospital between 1 April 2016 and 31 March 2019.

#### Patient-level outcomes

For each patient with an index hip fracture, all HES APC, outpatient clinic and emergency department (ED) attendance data in England, and similar PEDW data in Wales, will be analysed for the subsequent 12 months enabling post-fracture follow-up (thus the last follow-up will complete 31 March 2020). Patient outcome measures will include: (i) cumulative mortality at 30 days and 1 year, (ii) acute NHS ‘super-spell’ (defined as the index hip fracture admission, plus if applicable, planned hospital transfers for elective care and/or subsequent unplanned hospital transfers for emergency care), (iii) return to original residence at discharge, (iv) emergency 30-day readmissions (defined as an emergency all-cause admission to any English/Welsh NHS hospital that occurred within 30 days of hospital discharge following a hip fracture superspell), (v) mobility at 120 days, (vi) return to original residence at 120 days, (vii) osteoporosis treatment to reduce future fracture risk, (viii) re-fracture/re-operation, (ix) the total number of days spent in hospital in the year following hip fracture, informing (x) total direct health costs attributable to hip fracture (see below).

#### Health cost outcomes

HES data reports Healthcare Resource Groups assigned to each finished consultant episode in a hospital spell via the Casemix Grouper Software (HRG4+).^24^ HRGs are standard groups of clinically similar treatments that consume a common set of healthcare resources. HRGs will be valued using the most up-to-date prices available from Department of Health and Social care reference costs for NHS trusts, including a per diem costing for bed days in excess of those expected for a standard tariff.^25^

#### Statistical analysis of outcomes

**Figure 2:**
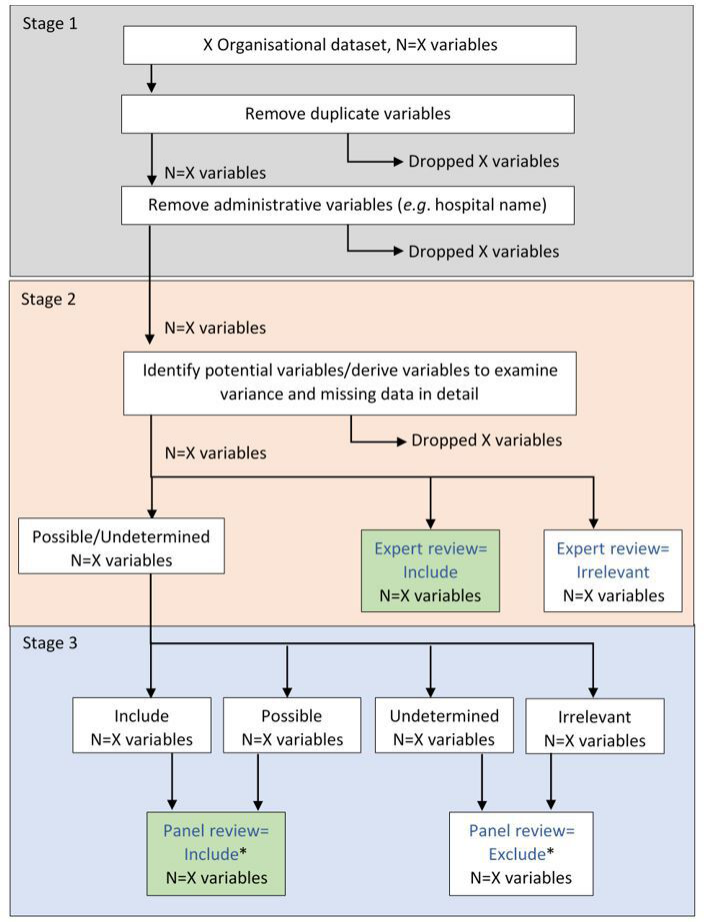
Flow diagram used to determine organisational data metrics. *Include if two or more of the four reviewers categorised variable as ‘include’ or ‘possible’, otherwise exclude; any lack of consensus resolved by a third reviewer (the principal investigator). Shaded boxes indicate variables which will be included in analyses.

Using a systematic approach, organisational factors will be identified which predict patient-level outcomes including associated health costs. Using a ‘top-down’ approach, defining a priori indicator groupings (domains), informed by NHFD BPT variables with stakeholder consensus, key indicators will be identified predicting patient outcomes. Clinicians will undertake expert panel review to select potential explanatory organisational-level variables. The flow diagram (figure 2) illustrates this review process which will be repeated across all organisational data sources.

**Figure 3:**
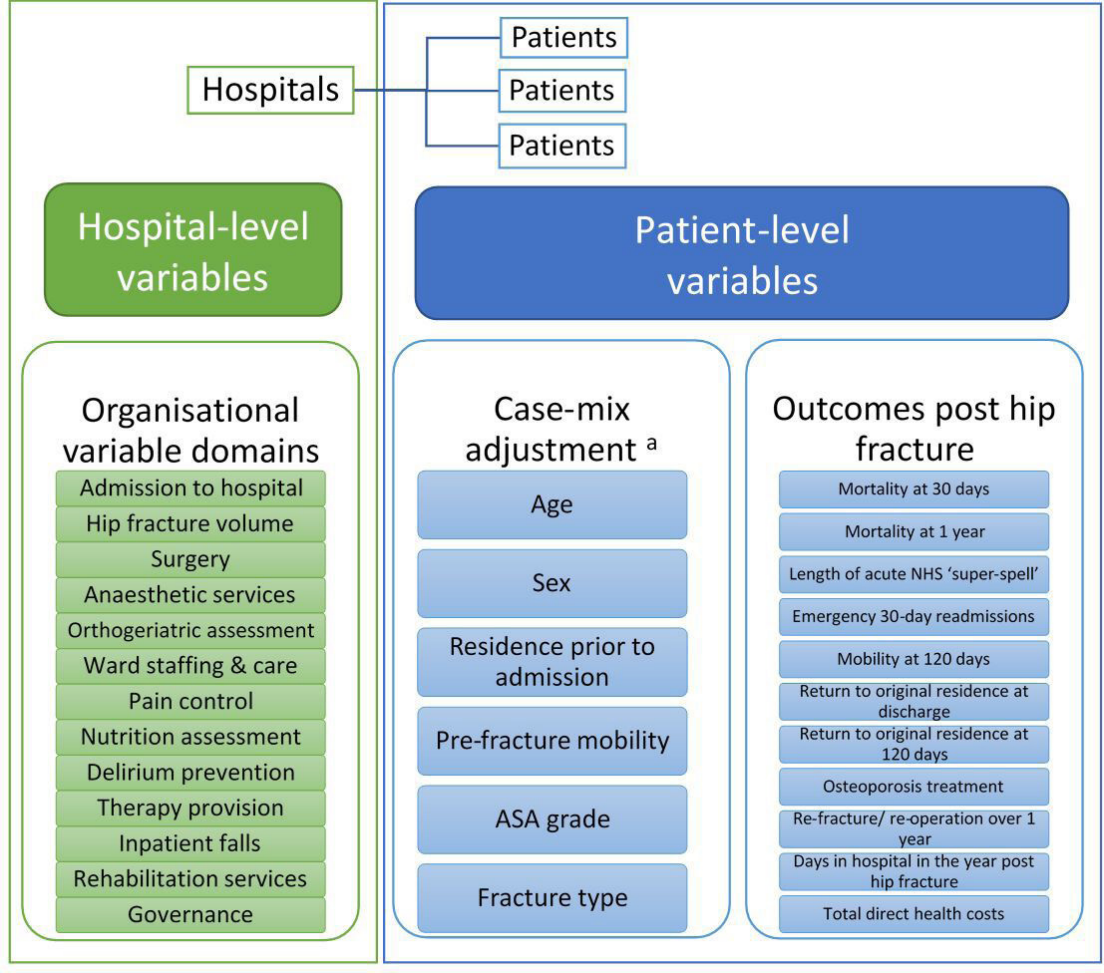
Structure of the multi-level models used in the statistical analyses in the REducing unwarranted variation in the Delivery of high qUality hip fraCture services in England and Wales study. ^a^Same as patient-level National Hip Fracture Database clinical audit case-mix variables.^26^ ASA, American Society of Anaesthesiologists.

Further expert-driven data reduction will involve examining the prevalence and correlation of selected organisational variables with each outcome, to finalise the dataset for multi-level models. Those variables selected for inclusion will be mapped to one or more domains of hip fracture care (eg, admission, anaesthesia, delirium prevention, governance, annual hospital admissions for hip fracture, inpatient falls, nutrition, orthogeriatrician assessment, pain management, rehabilitation, surgery, therapy provision and ward staffing and care (see figure 3). Each organisational variable will be assigned as relevant to one or more patient outcomes.

Multi-level regression modelling will identify organisational components of the hip fracture care pathway responsible for the greatest variation in patient outcomes and costs. The hierarchical data structure consists of patients (level 1), nested within hospitals (level 2). Multilevel regression models will describe the association of organisational-level factors on patient-level outcomes, while adjusting for patient case-mix, and allow assessment of interactions between patient-level and organisational-level factors. Case-mix adjustment will be the same as that used in the NHFD clinical audit^26^ and will include age, sex, residence prior to admission, pre-fracture mobility, fracture type and American Society of Anaesthesiologists grade^27^ (figure 3). Further adjustment will explore inclusion of the following additional patient-level variables as part of the case-mix adjustment: area-level deprivation, quantified by the Index of Multiple Deprivation^28^; comorbidity, quantified by the Charlson comorbidity index^29^ and frailty, quantified by the hospital frailty risk score.^30^

Having identified the proportion of variance in a given patient-level outcome, not explained by patient-level factors (ie, case-mix), the between-hospital variability explained by fixed organisational effects will be quantified, that is, the proportion of between-hospital variance explained by the configuration of specific services. For each outcome, domains added sequentially to a multi-level model (after case-mix adjustment) will be fitted to identify those domains which have the greatest effect and predict patient-level outcomes. From the selected domains the most influential organisational variables will be identified.

#### Statistical analysis of costs

Patient costs will be adjusted for baseline costs (ie, the healthcare costs incurred in the year prior to hip fracture) and patient case-mix as detailed earlier. Adjusted estimates of patient costs will be reported as aggregate costs and costs disaggregated by initial hospital superspell and readmissions/further NHS care. Different pathways of care following hip fracture will be identified by expert consensus agreement for comparative cost analyses.

The multi-level model will be used to determine how health costs relate to organisational factors and patient outcomes, identifying organisational factors associated with highest/lowest costs. Cost profiles will be calculated for different hip fracture care pathway models, facilitating budget impact analyses (eg, estimate potential NHS savings should hip fracture care pathway models change). Costed scenarios will inform the Implementation Toolkit cost-benefit calculator.

### Qualitative study

#### Service delivery stakeholder interviews

For the qualitative interviews four hospitals will be selected and approached. For English hospitals, BPT (2017–2019) and Key Performance Indicators (2018) will be collated, and 25 hospitals with the most and 25 with the least variable scores selected.^31^ Hospitals will be excluded if (i) they are major trauma centres (as these hospitals may differ significantly from the majority of hip fracture hospitals which are not major trauma centres); (ii) they have recently merged or moved location; (iii) they are participating in the hip fracture quality improvement programme (as practices in these hospitals are likely to be changing); (iv) they are located local to the study team (eg, Bristol, Bath) (to avert any conflict of interest within the research team); (v) if data quality is poor (as measured by NHFD report^1^); (vi) or if the hospital manages low volumes of hip fractures (annual hip fracture admissions less than the fifth percentile). From the remaining pool, hospitals will be selected to provide a diverse range in terms of 30-day mortality trend (2017–2018), BPT trend (2017–2019), hospital size (large/medium) and geographical location (eg, city/rural/coastal, north/south of England).

A qualitative interview study will characterise the organisation of hip fracture services and identify barriers and facilitators to the implementation of key components of fracture care. In addition, strategies that hospitals used to adapt and innovate hip fracture care delivery, during the COVID-19 pandemic, along with barriers and facilitators to the reorganisation of services in 2020 will be ascertained. Four hospitals have been identified that encompass variation in a range of characteristics as listed above, aiming to identify varied service configurations.^32^ Studying care in these settings will enable us to capture the experiences of those delivering different models of hospital care.

At the four participating hospitals, 1:1 in-depth interviews will be conducted with stakeholders involved in the organisation and delivery of hip fracture services, including orthogeriatricians, orthopaedic surgeons, anaesthetists, emergency medicine physicians, physiotherapists, occupational therapists, trauma nurses and discharge and service managers. Interviews will be carried out either remotely or in person (according to infection control constraints) with informed consent, including consent to audio-recording. An estimated 10–15 professionals will be interviewed at each hospital site, totalling around 40–60 professionals across the study. However, final sample size will be determined when data saturation is achieved; that is, when no new themes or subthemes are identified in the data.^33^ Interviews will be conducted using a topic guide with a list of themes and subthemes to guide discussions. This will enable us to compare and contrast stakeholder views and provide flexibility to pursue emerging ideas.^34^ To understand contextual factors that impact on service implementation, study design and analysis will be informed by Implementation Science. Implementation Science comprises theories or frameworks that have been used to help understand factors that help or hinder the delivery of complex interventions such as hip fracture care.^35–38^ The topic guide has been devised by the study team with Patient and Public Involvement (PPI). PPI identified patient priorities during the first wave of COVID-19, which will enable exploration of how services adapted to meet these needs. Four to six pilot interviews will inform topic guide refinement. If refinements are minor, initial pilot interviews will contribute to the main analysis.

Interviews will be audio-recorded, transcribed and anonymised. Transcripts will be imported into NVivo qualitative software and analysed using an inductive thematic analysis to identify key themes and subthemes in the data.^39^ Following this, an abductive approach will be used whereby themes/subthemes will be transposed onto concepts from Implementation Science theory.^40^ To ensure rigour, 20% of transcripts will be independently analysed in duplicate and themes reviewed and refined to agree a themes list.

### Documentary analysis of BOA reports

To complement the qualitative interviews and to understand the common themes and solutions (‘lessons learnt’) in relation to provision of hip fracture care, qualitative content analysis will be conducted of anonymised detailed hospital-initiated peer-review process (PRP) reports from 22 hospitals (all that have been conducted by the BOA to 2019). PRP reports, produced by the BOA over the period 2013–2019, were delivered when UK hospital service leads requested a BOA ‘peer-review’ to improve their hip fracture service. Each PRP report includes interviews with a range of staff (eg, clinical directors, clinicians, nurses, therapists, anaesthetists, ED personnel and managers). PRP reports are structured encompassing appraisal of the full hip fracture care pathway: ED, orthopaedics, anaesthetics, theatre activity, orthogeriatrics, nursing, therapies, discharge planning, collection of NHFD audit data and governance structures. Reports list all areas of good practice and highlight issues where improvements are achievable. PRP reports include a series of recommendations made by the multi-disciplinary assessment team with numerated action points. Reports will be imported into NVivo qualitative analysis software and will be analysed thematically to identify barriers and facilitators to implementation of quality hip fracture services.^39^ Themes identified in the documentary analysis will be mapped onto those from the qualitative interviews. To illustrate this process, data will be displayed on charts using the framework approach to data organisation.^41^ Written accounts will then be generated. Recommendations will inform choice of domains in quantitative analyses and of subsequent Implementation Toolkit development.

### Implementation Toolkit development

In 2015 the Royal (formerly National) Osteoporosis Society (ROS), developed a Fracture Liaison Service (FLS) Toolkit to aid FLS commissioning of new or improved services.^42^ It was designed to support business case development, saving time for service leads and commissioners, and has been highly successful. Since 2015 the Service Improvement Team at the ROS have supported the development of 34 new FLSs. These new services cover a patient population of more than 12 million people and it has been estimated that 5 years following implementation of all these services, approximately 3854 hip fractures will have been prevented. Approximately 60% of the UK population can access an FLS, and the ROS is currently working with sites all over the UK to ensure that current services are delivering in line with national clinical guidelines, and supporting sites that do not have an FLS at present. The ROS has acquired extensive experience in service improvement, refining tools based on user feedback, and continuing to support quality improvement.

Working with the ROS, the BOA and other stakeholders, a Hip Fracture Implementation Toolkit will be codeveloped, focusing on inpatient hip fracture services, prioritising organisational factors identified from our quantitative and qualitative results which contribute to poor and/or highly variable patient outcomes post hip fracture. All acute NHS hospitals currently have a hip fracture care pathway; hence, the toolkit will include a step-by-step guide to improve and implement changes to current services. It will be made available online and provide a series of instructions and guides (ie, ‘tools’) for managers, clinical leads and healthcare system leads to use to improve their hospital hip fracture services, encompassing service redesign/restructuring, organisational cultural change, and approaches to improve efficient use of limited healthcare resources. Tools will include a service improvement guide, business case resources, a cost calculator and a project plan.

### Patient and public involvement

This study has been developed in collaboration with the University of Bristol Musculoskeletal Research Unit (MRU) PPI group comprising members who have had/are having treatment for osteoporosis and/or fractures, who meet (currently virtually) every 3 months to input into the design and conduct of MRU research projects. They have guided development of the project proposal, informing prioritisation of research questions, drafting plain English text, changing language and wording within study texts; they have informed methods adaptation in response to COVID-19 in 2020. The group will provide ongoing support throughout the study, addressing (i) key patient and carer questions and priorities, (ii) the interpretation and relevance of results and (iii) communication of findings. ‘Taking the research’ to patients in the involvement group rather than asking patients to attend research management meetings has improved engagement and fosters strong collaboration and respect.^43^ Meetings will be organised by an experienced PPI coordinator (KW), who will facilitate meetings, provide ongoing support and tailored development to patient members, and advise on good practice.

## Ethics and dissemination

### Ethics and governance

The quantitative study has research approvals from NHS Health Research Authority—London City and East Research Ethics Committee (20/LO/0101, 11/02/2020); Royal College of Physicians (RCP) Falls and Fragility Fracture Audit Programme (FFFAP) (FFFAP/2018/003, 11/12/2019) and Healthcare Quality Improvement Partnership approval (HQIP330, 18/06/2020); NHS Wales Informatics Service (NWIS) (30941, 13/03/2020) and an NHS Digital Data Sharing Agreement (DARS-NIC-334549-B1Y6X-v1.4, 28/09/2020). The qualitative study has been approved for conduct by the Faculty of Health Sciences University of Bristol Research Ethics Committee (Ref: 108284, 9/9/2020) and by the NHS Health Research Authority (20/HRA/71, 10/9/2020). Each NHS Trust has provided Research and Development approval.

### Dissemination

Findings will be disseminated through scientific conferences, peer-reviewed publications and online implementation workshops. Results will be fed back to the scientific committee with oversight of the NHFD as well as the BOA. Reports will be provided to each hospital in England and Wales summarising findings, and the PPI group will be involved in all stages of dissemination. Working with the ROS, dissemination materials will be developed for its membership network (n=20 000). The Implementation Toolkit will be hosted and made freely available by the ROS website.

## Conclusion

Through the use of mixed methodology, this study will determine the components of hospital service delivery which account for poor patient outcomes post hip fracture and identify which service configurations are most efficient and successful. Quantitative analyses will allow us to distinguish patient outcomes explained by the health of the patient themselves, versus outcomes attributable to the hospital services which they encounter. Domains of hip fracture care which are most critical for a wide range of patient outcomes over 12 months, will be identified. The direct health costs ascribed to each patient in the year after hip fracture, accounting for costs in the year prior to fracture, will be calculated. Thus, hospital expenditure attributable to different components of hip fracture service delivery will be calculated. These financial calculations will inform cost calculators in the Implementation Toolkit. The Toolkit will be a novel, freely available online resource for managers, clinical leads and healthcare system leads to use to improve their hip fracture service.

The qualitative analysis will aid understanding of the organisational processes that help or hinder the implementation of key components of hip fracture services. In addition, it will identify strategies that hospitals have used to adapt and innovate hip fracture care delivery during the COVID-19 pandemic. Knowledge gained will inform toolkit development, aiming to assist services in overcoming organisational barriers when designing and implementing sustainable high-quality fracture services, and improve patient care. Understanding strategies hospitals used to reconfigure care during the COVID-19 pandemic will provide learning towards the development of more robust and resilient hip fracture services in the future. This information is likely to be transferrable to other services.

In conclusion, a system-wide understanding of sources of variation in hip fracture care delivery and the effects on patient outcomes will inform service-level interventions to reduce unwarranted variation, maximise health equity and ultimately improve patient experience. The study aims to show effective hip fracture care is more efficient, realising cost-savings in hospital bed-days potentially re-directable to other services. This project is novel in terms of its scale and the unique datasets which gives us a rare opportunity to robustly assess what is a complex system of care and the very real impacts this system has on patients. Findings will inform future commissioning/service planning priorities for hip fracture care, inform national review processes for hip fracture services and, together with a new online toolkit, this programme aims to minimise avoidable variation in hip fracture care and improve the quality of care for patients across the UK.

## Supplementary Material

Reviewer comments

Author's manuscript
