# Supplementary material for: REducing unwarranted variation in the Delivery of high qUality hip fraCture services in England and Wales (REDUCE): protocol for a mixed-methods study
Source: BMJ Open. 2021 May 19;11(5):e049763. doi: 10.1136/bmjopen-2021-049763 (PMC8137248; doi:10.1136/bmjopen-2021-049763)
Supplement: Reviewer comments [file bmjopen-2021-049763.reviewer_comments.pdf]

PEER REVIEW HISTORY

BMJ Open publishes all reviews undertaken for accepted manuscripts. Reviewers are asked to complete a checklist review form (**http://bmjopen.bmj.com/site/about/resources/checklist.pdf**) and are provided with free text boxes to elaborate on their assessment. These free text comments are reproduced below.

ARTICLE DETAILS

**TITLE (PROVISIONAL)**	Reducing unwarranted variation in the delivery of high quality hip fracture services in England and Wales (REDUCE): protocol for a mixed-methods study
**AUTHORS**	Patel, Rita; Drew, Sarah; Johansen, Antony; Chesser, Tim; Javaid, Kassim; Griffin, Xavier; Jones, Tim; Griffin, Jill; Bradshaw, Marianne; Whale, Katie; Barbosa, Estela; Marques, Elsa; Ben-Shlomo, Yoav; Gooberman-Hill, Rachael; Judge, Andrew; Gregson, Celia

This article was not externally reviewed at BMJ Open. Protocols that have been independently assessed prior to submission to BMJ Open are usually fast-tracked to publication on the grounds that further substantial changes will not be possible. This independent assessment will usually be external, independent review for both a substantial grant award from a non-commercial or government funder and ethics approval.
